# Health Attitudes, Health Cognitions, and Health Behaviors among Internet Health Information Seekers: Population-Based Survey

**DOI:** 10.2196/jmir.6.2.e15

**Published:** 2004-05-28

**Authors:** Mohan J Dutta-Bergman

**Affiliations:** ^1^Department of CommunicationPurdue UniversityUSA

**Keywords:** Internet, health beliefs, health consciousness, consumers, information seeking, functional approach

## Abstract

**Background:**

Using a functional theory of media use, this paper examines the process of health-information seeking in different domains of Internet use.

**Objective:**

Based on an analysis of the 1999 HealthStyles data, this study was designed to demonstrate that people who gather information on the Internet are more health-oriented than non-users of Internet health information.

**Methods:**

The Porter Novelli HealthStyles database, collected annually since 1995, is based on the results of nationally representative postal mail surveys. In 1999, 2636 respondents provided usable data for the HealthStyles database. Independent sample t-tests and logistic regression analyses were conducted.

**Results:**

The results showed that individuals who searched for health information on the Internet were indeed more likely to be health-oriented than those who did not. Consumers who sought out medical information on the Internet reported higher levels of health-information orientation and healthy activities, as well as stronger health beliefs than those who did not search for medical news on the Internet. It was observed that those who reported searching for information about drugs and medications on the Internet held stronger health beliefs than the non-searchers. Comparison of individuals who reported seeking out information about specific diseases on the Internet with individuals who did not showed those who sought out disease-specific information on the Internet to be more health-oriented. Finally, consumers who sought out healthy lifestyle information on the Internet were more health conscious and more health-information oriented than those who did not. They were also more likely to hold stronger health-oriented beliefs and to engage in healthy activities.

**Conclusions:**

The results support the functional theory of Internet use. Internet searchers who used the Internet for a wide range of health purposes were typically more health oriented than non-searchers.

## Introduction

The exponential growth in health-care consumerism and limitless consumer access to health information have propelled a surge in scholarship on eHealth [1]. In the last few years, the Internet has become central to the process of health-based consumer decision-making, resulting in a tremendous growth in expert debates about the Internet's impact on the health-care consumer [2]. Although the extant health literature supports the existence of systematic motivational differences in health orientations, consumer use of the Internet has not yet been interrogated in the context of health motivation [3,4,5]. This article examines the idea that the motivational differences in health orientation drive consumer search for health information on the Internet [6,7]. Based on a functional approach to Internet use, the article argues that the health-oriented consumer is more likely to seek out a variety of health-based information on the Internet than people who are not health-oriented [8,9].

The functional approach to media use posits that people use a given medium for many different reasons [10]. The function served by a particular medium emerges from the communicative needs of the audience [11]. Communication behavior, in the functional realm, is goal-directed, and individuals select and use communication channels to satisfy felt needs [12]. This article examines consumer behavior in the following information functions of the Internet: (a) gathering medical news, (b) looking for information about medical services, (c) searching for information about drugs and medications, (d) gathering disease-specific information, (e) searching for information about healthy lifestyle, and (f) looking for discussion groups. It uses the HealthStyles data to examine the differences in demographic, attitudinal, and cognitive variables between individuals on the basis of the different Internet sources of health information that they consider to be most credible.

## Methods

### Data

The Porter Novelli HealthStyles database, collected annually since 1995, is based on the results of three postal mail surveys. The initial survey, the DDB Needham Lifestyles survey (commissioned by DDB Needham Worldwide), is sent to a stratified random sample of approximately 5000 US adults in April of each year. The sample is generated from a panel of 500,000 cooperating households that represent a range of sociodemographic characteristics. Approximately 3400 responses were obtained for the 1999 Lifestyles survey, representing a response rate of 68%. The second survey is a supplemental mailing of the Lifestyles survey to adjust the representation of particular households in the database. In 1999, the supplemental mailing was sent to 210 low-income households and 210 minority households to compensate for their lower return rates. The third survey, HealthStyles, is sent to respondents who complete either the initial or supplemental Lifestyles survey. Respondents to each of the surveys are sent small gifts for their participation (such as a 20-minute calling card) and are entered into a cash prize drawing. In 1999, 2636 (74%) respondents provided usable data for the Healthstyles survey. The entire sample is weighted on age, sex, race/ethnicity, income, and household size to reflect the US Census population. The demographic comparison of the sample with the 2000 Census data is provided in Table 1.

**Table 1:** HealthStyles 1999 data comparison with 2000 Census data (medians or %)

Category		1999 HealthStyles Data	2000 Census Data
Mean Age		42	35
Gender	Men	48%	49%
	Women	52%	51%
Race	White	74.6%	77%
	Black	11.6%	12.3%
	Other	13.8%	10.7%

### Measures

#### Health-information Functions

To measure the different online health-information functions engaged in by the consumer, the following guideline was provided: "When looking for information on the Internet, which topics do you mainly look for?" Categories included (a) medical news, (b) medical services, (c) drugs and medications, (d) specific diseases, (e) how to stay healthy, and (f) discussion groups on health. Responses were measured in a dichotomous Yes/No format.

#### Health Consciousness

Health consciousness was measured by five items on a 1 to 5 scale with 1 representing "strongly disagree," and 5 representing "strongly agree." When subjected to a principal axis factor analysis, a single factor was produced with an Eigenvalue of 2.36 and explaining 47.24% of the variance (see Table 2). The Cronbach alpha for the scale was .72.

**Table 2:** Principal axis analysis of health consciousness attitude

Items	Loading	α
I do everything I can to stay healthy.	.76	
Living life in best possible health is very important to me.	.74	
I actively try to prevent disease and illness.	.73	
Eating right, exercising, and taking preventive measures will keep me healthy for life.	.62	
My health depends on how well I take care of myself.	.57	
		.72

#### Health-information Orientation

Eight items were used to measure health-information orientation on a 1 to 5 scale. A principal axis factor analysis produced a single factor with an Eigenvalue of 4.18 (see Table 3). Factor loadings ranged from .55 to .80 and the factor explained 52.24% of the variance. Cronbach alpha for the aggregated scale was .87.

**Table 3:** Principal axis analysis of health-information orientation

Items	Loading	α
It's important to me to be informed about health issues.	.82	
I need to know about health issues so I can keep myself and my family healthy.	.80	
Before making a decision about my health, I find out everything I can about the issue.	.73	
I really enjoy learning about health issues.	.73	
To be and stay healthy it's critical to be informed about health issues.	.71	
When I take medicine, I try to get as much information as possible about its benefits and side effects.	.68	
I make a point to read and watch stories about health.	.68	
The amount of health information available today makes it easier for me to take care of my health.	.62	
		.87

#### Health-oriented Beliefs

The respondents were provided the following instruction: "please rate each of the following health behaviors on a scale of 1 to 5 depending on how important you think that behavior is for your overall health." Items included "eating a diet that is low in fat," "eating lots of fruits, vegetables, and grains," drinking plenty of water every day," "taking vitamins and mineral supplements regularly," "exercising regularly," "not smoking cigarettes," "not drinking alcohol or drinking in moderation," and "maintaining a healthy body weight." Cronbach alpha for the aggregated scale was .82.

#### Healthy Activities

Healthy activities were measured by eight items. The respondents were provided the following instruction: "please place an X for each of these behaviors that you currently perform to maintain your health." Items included "eating a diet that is low in fat," "eating lots of fruits, vegetables, and grains," drinking plenty of water every day," "taking vitamins and mineral supplements regularly," "exercising regularly," "not smoking cigarettes," "not drinking alcohol or drinking in moderation," and "maintaining a healthy body weight." Responses were measured on a dichotomous Yes/No format, and the activities were summed up to constitute the healthy activities variable. It is important to note that the scale used to measure health activities is different from the scales used to measure health consciousness, health-information orientation and health-oriented beliefs.

### Data Analyses

The data were entered into Statistical Package for the Social Sciences (SPSS 10.0). Correlation analysis, t-tests and a binary logistic regression were run to analyze the data. Six information seeking functions were identified: (a) medical news, (b) medical services, (c) drugs and medications, (d) specific disease, (e) healthy lifestyle, and (f) discussion group. For each information-seeking function, a t-test was conducted in each of the four areas: health consciousness, health-information orientation, health beliefs, and health activities.

## Results

Correlation analysis demonstrated that the independent variables were positively correlated with one another at the p<.001 level (see Table 4).To analyze the relationship between Internet functions and health-oriented variables, independent sample t-tests were conducted. Given that four t-tests (attitudes, information orientation, beliefs, and activities) were conducted for each information function on the Internet, Bonferroni correction was used to adjust the alpha level by the number of tests. The adjusted alpha for each of the hypotheses was .05/4 = .0125.

**Table 4:** Correlation among health consciousness, health-information orientation, health beliefs, and health activities

Variables				
Health Consciousness	1.00			
Health-information Orientation	.62*	1.00		
Health Beliefs	.46*	..45*	1.00	
Health Activities	.32*	.27*	.46*	1.00

^*^ p < .001

### Internet Health-information Use

The results presented in Table 5 show that individuals who searched for health information on the Internet were indeed more likely to be health conscious and health-information oriented, hold strong health beliefs, and engage in healthy activities than individuals who did not search for health information on the Internet.

**Table 5:** Comparison of health consciousness, health-information orientation, health beliefs, and health activities in the context of Internet health-information use

Health-oriented Variables	Internet Health Information		t	α ^2^
	Searcher (n = 979)	Non-searcher (n = 1657)		
Health Consciousness	3.95 (SD = .64)	3.95 (SD = .66)	.05	.003
Health-information Orientation	3.80 (SD = .68)	3.61 (SD = .75)	6.24*	.008
Health-oriented Beliefs	4.23 (SD = .57)	4.08 (SD = .74)	5.14*	.004
Healthy Activities	4.19 (SD = 2.34)	3.66 (SD = 2.50)	5.41*	.009

^*^ p < .001; Bonferroni correction was conducted and significance level was set at .05/4 = .0125.

### Medical News Seeking

Table 6 presents the comparison between individuals who sought out medical news on the Internet with individuals who did not use the Internet to look for medical news. The results show that consumers who sought out medical information on the Internet reported higher levels of health-information orientation and healthy activity, and stronger health beliefs than those respondents who did not search for medical news on the Internet. However, no significant differences were observed between searchers and non-searchers for medical news on the Internet in the realm of health consciousness.

**Table 6:** Comparison of health consciousness, health-information orientation, health beliefs, and health activities in the context of medical news use

Health-oriented Variables	Medical News		t	α ^2^
	Searcher (n = 345)	Non-searcher (n = 2291)		
Health Consciousness	4.00 (SD = .69)	3.94 (SD = .65)	1.52	.001
Health-information Orientation	3.91 (SD = .69)	3.65 (SD = .73)	6.24*	.013
Health-oriented Beliefs	4.26 (SD = .58)	4.11 (SD = .69)	3.60*	.005
Healthy Activities	4.37 (SD = 2.28)	3.78 (SD = 2.47)	4.16*	.007

^*^ p < .001; Bonferroni correction was conducted and significance level was set at .05/4 = .0125.

### Medical Service Information

In the domain of searching for medical service information on the Internet, the t-tests demonstrated no significant differences between searchers and non-searchers in the realms of health consciousness, health-oriented beliefs, and healthy activities. A significant difference was found only in the realm of health-information orientation, with searchers being more likely to be health-information oriented than non-searchers.

**Table 7:** Comparison of health consciousness, health-information orientation, health beliefs, and health activities in the context of medical services

Health-oriented Variables	Medical Services		t	α ^2^
	Searcher (n = 105)	Non-searcher (n = 2531)		
Health Consciousness	3.82 (SD = .79)	3.96 (SD = .65)	1.99	.001
Health-information Orientation	3.96 (SD = .73)	3.67 (SD = .73)	3.88*	.006
Health-oriented Beliefs	4.24 (SD = .55)	4.13 (SD = .69)	1.62	.001
Healthy Activities	4.33 (SD = 2.34)	3.83 (SD = 2.46)	2.00	.001

^*^ p < .010

p < .001; Bonferroni correction was conducted and significance level was set at .05/4 = .0125.

### Drug and Medication Information

In the realm of consumer information search for information about drugs and medications on the Internet, it was observed that searchers held stronger health beliefs than the non-searchers (see Table 8). Searchers were also more likely to be health-information oriented and engage in healthy activities than non-searchers. However, no significant differences were observed between searchers and non-searchers in the realm of health consciousness.

**Table 8:** Comparison of health consciousness, health-information orientation, health beliefs, and health activities in the context of seeking information about drugs and medications

Health-oriented Variables	Drugs and Medications		t	α ^2^
	Searcher (n = 382)	Non-searcher (n = 2254)		
Health Consciousness	3.96 (SD = .67)	3.95 (SD = .65)	.45	.000
Health-information Orientation	3.93 (SD = .66)	3.64 (SD = .73)	7.27*	.017
Health-oriented Beliefs	4.25 (SD = .58)	4.11 (SD = .70)	3.51*	.004
Healthy Activities	4.26 (SD = 2.34)	3.79 (SD = 2.47)	3.51*	.007

^*^ p < .001; Bonferroni correction was conducted and significance level was set at .05/4 = .0125.

### Disease-specific Information

Table 9 compares individuals who reported seeking out information about specific diseases on the Internet with individuals who did not seek out disease-specific information on the Internet. Differences were observed in the realms of health-information orientation, health beliefs, and healthy activities, with those who sought out disease-specific information on the Internet being more health-oriented than those who did not. However, no significant differences were observed between disease-specific health-information seekers and non-seekers in the domain of health consciousness.

**Table 9:** Comparison of health consciousness, health-information orientation, health beliefs, and health activities in the context of seeking information about specific diseases

Health-oriented Variables	Specific Diseases		t	α ^2^
	Searcher (n = 619)	Non-searcher (n = 2017)		
Health Consciousness	3.97 (SD = .61)	3.94 (SD = .67)	.95	.003
Health-information Orientation	3.84 (SD = .66)	3.64 (SD = .74)	6.23*	.013
Health-oriented Beliefs	4.23 (SD = .55)	4.10 (SD = .72)	4.07*	.005
Healthy Activities	4.27 (SD = 2.27)	3.73 (SD = 2.50)	4.77*	.007

^*^ p <.001; Bonferroni correction was conducted and significance level was set at .05/4 = .0125.

### Healthy Lifestyle Information

Consumers who sought out healthy lifestyle information on the Internet were more health conscious and more health-information oriented than those other consumers who did not seek out health information (see Table 10). They were also more likely to hold stronger health-oriented beliefs and to engage in healthy activities.

**Table 10:** comparison of health consciousness, health-information orientation, health beliefs, and health activities in the context of seeking healthy lifestyle information

Health-oriented Variables	Staying Healthy		t	α ^2^
	Searcher (n = 272)	Non-searcher (n = 2364)		
Health Consciousness	4.09 (SD = .61)	3.93 (SD = .66)	3.62*	.003
Health-information Orientation	3.99 (SD = .61)	3.65 (SD = .73)	7.45*	.018
Health-oriented Beliefs	4.36 (SD = .48)	4.11 (SD = .70)	5.64*	.010
Healthy Activities	4.71 (SD = 2.22)	3.75 (SD = 2.46)	6.09*	.012

^*^ p <.001; Bonferroni correction was conducted and significance level was set at .05/4 = .0125.

### Health-based Discussion Groups

Individuals who sought out health-based discussion groups on the Internet were more likely to be health-information oriented than individuals who did not seek out discussion groups on the Internet (see Table 11). No significant differences were observed in the realms of health consciousness, health beliefs, and healthy activities.

**Table 11:** Comparison of health consciousness, health-information orientation, health beliefs, and health activities in the context of seeking discussion groups

Health-oriented Variables	Discussion Groups		t	α ^2^
	Searcher (n = 37)	Non-searcher (n = 2599)		
Health Consciousness	4.16 (SD = .57)	3.95 (SD = .66)	1.95	.001
Health-information Orientation	4.02 (SD = .72)	3.67 (SD = .73)	2.83*	.002
Health-oriented Beliefs	4.25 (SD = .64)	4.13 (SD = .68)	.10	.000
Healthy Activities	4.61 (SD = 2.45)	3.84 (SD = 2.45)	1.89	.001

^*^ p < .005; Bonferroni correction was conducted and significance level was set at .05/4 = .0125.

### Health-oriented Variables and Internet Functions

Finally, to account for the correlation among the different health-oriented variables, six logistic regression analyses were conducted (see Table 12). The health-oriented variable that consistently demonstrated the strongest relationship with the different Internet information functions was health-information orientation.

**Table 12:** The relationship between health-oriented variables and Internet functions

	Medical News		Medical Services		Drugs and Medications		Specific Disease		Healthy Lifestyle		Discussion Group	
	B	P	B	P	B	P	B	P	B	P	B	p
Hlth Csnss	-.46	<.001	-1.3	<.001	-.74	<.001	-.45	<.001	-.27	.06	.21	.59
Hlth Beliefs	.08	.50	.11	.58	.09	.44	.09	.32	.25	.08	-.27	.41
Hlth Inf. Orntn	.70	.001	1.21	<.001	.97	<.001	.62	<.001	.72	<.001	.47	.17
Hlth Actvts	.07	.01	.09	.07	.05	.06	.07	.004	.10	.003	.12	.18
Nagelkerke R ^2^	.044		.074		.07		.045		.065		.024	

## Discussion

The results provided support for the functional approach to Internet consumption in the health context. Motivation emerged as a critical factor in driving consumption of media types. Demonstrating a match between motivation and content choice, health-orientation was positively correlated with information seeking on the Internet. The results suggest that the underlying motivation in a specific issue is likely to draw the consumer to use media (such as the Internet) to gather information about the specific issue. This match between content-based motivation and Internet content use is likely to be strong because of the user-driven nature of the Internet.

Consumers who sought out medical news on the Internet were more health conscious and health-information oriented, held stronger health beliefs, and were more likely to engage in healthy activities. In the domain of Internet use for gathering information about medical services, the only realm where systematic differences were observed between searchers and non-searchers was health-information orientation. Once again, this result makes sense in the context of the functional approach to the Internet. Searching the Internet for medical services information is a reflection of health-information orientation. Given the finding that the health-information-oriented consumer uses the Internet for procuring information about medical services, service providers should use the medium to reach out to health-oriented individuals.

Consumers who seek out information about drugs and medications on the Internet are also more health-information oriented. They hold stronger health beliefs and are more likely to engage in healthy activities. Pharmaceutical companies and providers of treatment options could effectively harness the ability of the Internet to reach the health-active group. The message, however, must be cogently constructed, and strong arguments must be provided because searchers are actively engaged in their health decisions. It is also important to present complete health information given the active orientation of the group. The search for disease-specific information was positively correlated with health orientation. Also, the search for information about a health lifestyle was positively associated with health consciousness, health-information orientation, health beliefs, and healthy activities. Developers of new health solutions should target this health-active group given its strong involvement with health information. Finally, individuals who sought out discussion groups on the Internet were more health-information oriented, although no other differences were observed.

The t-tests, and subsequently the regression analysis, pointed out that the strongest effect across the different Internet health-information functions is in health-information orientation. This is perhaps a result of the fact that health-information orientation is most closely aligned with specific health-information-seeking functions on the Internet. The negative relationship of health consciousness to the Internet information-seeking functions is attributable to the multicollinearity. This is proven by the results of the independent t-tests that demonstrated either no relationship or positive relationship between health-conscious attitude and Internet information-seeking function.

One of the limitations of the study was its use of self-reported measures. Self-reported indicators of health consciousness, health beliefs, health-information orientation, and healthy activities raise questions about validity. The "topics of health information" variable was treated as a dichotomous variable measured in a Yes/No format; therefore, it did not provide information about the degree of consumer use of the different health topics. The items "eating right, exercising, and taking preventive measures" and "eating lots of fruits, vegetables, and grains" were triple-barreled. The mailback panel used in the study suffers from problems of attrition and panel bias. Also, the effect sizes were typically small. Finally, the use of an American sample that is predominantly white limits the generalizability of the study results. Future research needs to extrapolate the research findings to other cultural domains.
